# Persistent *Cutibacterium* (Formerly *Propionibacterium*) *acnes* Bacteremia and Refractory Endocarditis in a Patient with Retained Implantable Pacemaker Leads

**DOI:** 10.1155/2020/8883907

**Published:** 2020-07-25

**Authors:** M. Freedman, J. O. Aflatooni, R. Foster, P. G. Haggerty, C. J. Derber

**Affiliations:** Internal Medicine, Division of Infectious Disease, Eastern Virginia Medical School, 825 Fairfax Avenue, Suite 461 Hofheimer Hall, Norfolk, VA 23507, USA

## Abstract

*Cutibacterium* (formerly *Propionibacterium*) *acnes* (*C. acnes*) is a commensal bacteria commonly found on the human skin and in the mouth. While the virulence of *C. acnes* is low in humans, it does produce a biofilm and has been identified as an etiologic agent in a growing number of implant-associated infections. *C. acnes* infections can prove diagnostically challenging as laboratory cultures can often take greater than 5 days to yield positive results, which are then often disregarded as contaminant. Patients with recurrent bacteremia in the setting of implantable devices warrant further studies to evaluate for an associated valvular or lead endocarditis. The patient in this report demonstrates how cardiac device-related endocarditis secondary to *C. acnes* can be overlooked due to the indolent nature of this pathogen. This patient presented with an implanted cardiac pacemaker device, as well as retained leads from a prior pacemaker. Transesophageal echocardiography was required to confirm the diagnosis in the setting of multiple positive blood cultures and negative transthoracic echocardiograms over a period of 4 years. The purpose of this report is to highlight the difficulties encountered in diagnosing *C. acnes* endocarditis in a patient with a cardiac implantable electronic device and persistently positive blood cultures.

## 1. Introduction

*C. acnes* is a Gram-positive anaerobe that is recognized as a part of the commensal flora of human skin. This nonmotile, non-spore-forming *Bacillus* is one of the predominant microorganisms of the dermatologic microbiota, especially in sebaceous gland-rich areas such as the chest, face, and scalp [1, 2]. While best known for its role in acne, recent studies have identified *C. acnes* as the etiologic agent in a growing number of implant-associated infections. Shoulder prosthetic joint infections and cerebrovascular shunt infections are the most common, but infections of cardiovascular devices are also being identified now [2]. It is hypothesized that the ability of certain strains of *C. acnes* to produce biofilms can lead to colonization of cardiac pacemaker devices without overt signs of clinical infection [3].

Diagnosing *C. acnes* as the causative source of infection can prove exceedingly difficult for a variety of reasons. Positive blood cultures may take more than 5 days to grow and are often disregarded as a contaminant; blood cultures in patients with deep-seated infections can be negative in up to one-third of cases. [4, 5] We present a case of pacemaker lead infection with *C. acnes* that was repeatedly either considered a skin contaminant or inadequately treated despite persistently positive blood cultures.

## 2. Patient History

**Figure 1:**
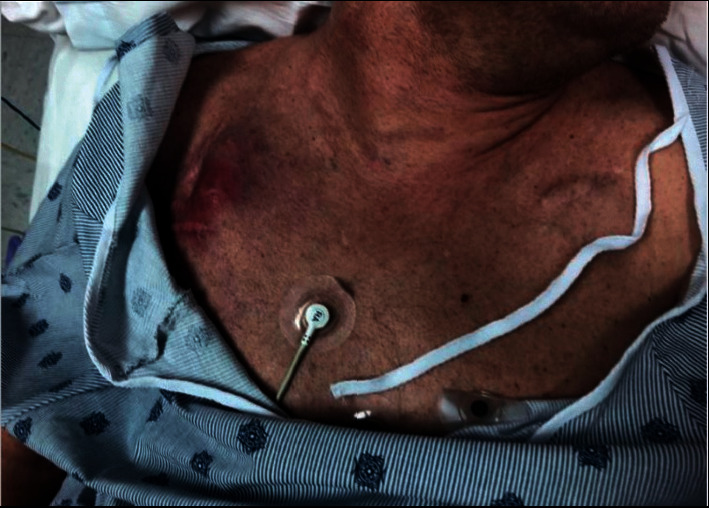
Patient's chest at initial presentation to the emergency department, with erythematous fluctuance at the pocket of his previous right-sided pacemaker visible.

A 52-year-old man with a past medical history significant for uncontrolled type 2 diabetes, remote traumatic brain injury, and complete heart block of unknown etiology presented to the emergency department (ED) due to symptomatic hypoglycemia, intermittent fevers and chills, and a swollen, erythematous, tender fluctuance at the pocket of his previous right-sided pacemaker that had been removed years ago, as seen in Figure 1. The patient had no documented history of pacemaker pocket infections.

In regards to his cardiac history, the patient had required extensive surgical management of his complete heart block. He had a permanent pacemaker (PPM) implanted at 22 years of age for this condition, which had since required three different surgeries for pulse generator and cardiac lead replacement and revision. At the time of his presentation to the ED, the patient had a dual chamber PPM in the left chest implanted 6 years prior; he also had the aforementioned pocket in his right chest, a potential space remaining after the removal of his previous PPM. Additionally, he had two retained ventricular leads from another previous pacemaker embedded in his myocardium, which had been trimmed back as much as possible and capped off, but never removed since they could not be safely extracted. The exact lengths of the retained leads were not discussed in the postoperative reports.

Due to high clinical suspicion for infection of the right-sided pocket, as well as concerns for sepsis, the patient was started on empiric antibiotic therapy (piperacillin/tazobactam and intravenous (IV) vancomycin). He was then taken to the operating room for incision and drainage (I&D) of the infected pocket site and placement of a wound vacuum. On hospital day (HD) 4, despite his I&D, wound vacuum, and antibiotic regimen, the patient was still having fevers and leukocytosis. Later that day, his blood cultures grew Gram-positive rods in both anaerobic bottles, and the infectious diseases service was subsequently consulted. Due to concern for infective endocarditis, a transthoracic echocardiogram (TTE) was ordered, though it did not show any evidence of heart failure, valvular abnormalities, or vegetations. On HD 6, *C. acnes* was isolated from the anaerobic blood cultures from admission and from intraoperative wound cultures taken from the right-sided pacemaker pocket. His antibiotic regimen was, then, narrowed to ceftriaxone 2 grams IV daily.

**Figure 2:**
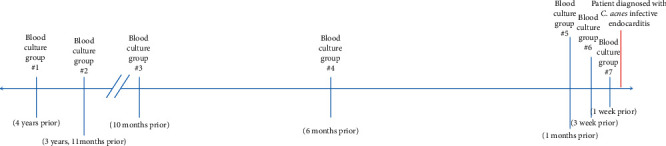
Each *C. acnes*-positive blood culture in relation to the time at which the patient was diagnosed with infective endocarditis.

Of note, the patient had six other recorded instances over the previous 4 years in which he had blood cultures drawn, all of which were initially positive for *C.* acnes. Per his notes, the majority of these cultures had either been attributed to contaminant or dismissed. If follow-up blood cultures were pursued, it was usually after the patient had received multiple days of empiric antibiotic therapy, making it difficult to interpret their negative results. An overview of the circumstances surrounding the patients most recent and six previous *C. acnes*-positive blood cultures, as well as a timeline of these blood cultures, can be seen in Table 1 and Figure 2, respectively. Also, over the same four-year period, the patient had four TTEs for various reasons, none of which showed any evidence of infective endocarditis. The patient never had a transesophageal echocardiogram (TEE) prior to this admission.

**Figure 3:**
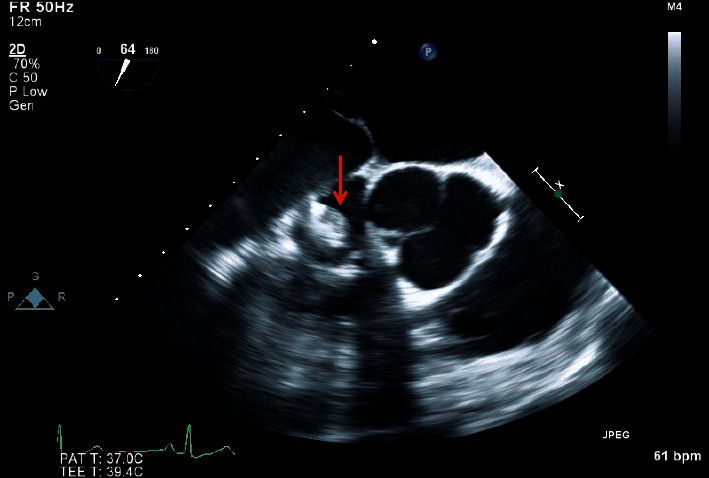
Transesophageal echocardiogram showing 1 cm vegetation (red arrow) adhered to the tricuspid valve suggestive of infective endocarditis.

Considering the positive wound and blood cultures from this hospitalization and his history of persistent *C. acnes*-positive blood cultures in the presence of an implantable cardiac device and retained leads, infective endocarditis was still suspected and a TEE was recommended. This study was performed on HD 9 and revealed vegetations across multiple pacemaker leads, highly suggestive of infective endocarditis. The largest vegetation was 1 cm in diameter, as seen in Figure 3.

Cardiothoracic surgery was consulted and, due to the infection, recommended removal of the left-sided dual chamber PPM, as well as extraction of the retained, embedded leads from the previous pacemaker. The left-sided PPM was removed without complication, but the retained leads were fractured and deeply embedded in the myocardium and, thus, could not be safely extracted again. Open thoracotomy to remove the embedded leads was offered; however, the patient opted for conservative management entailing 6 weeks of ceftriaxone, followed by indefinite suppression with oral doxycycline. He was discharged on HD 25 to a skilled nursing facility for further rehab and completion of his IV antibiotic therapy.

The patient was followed up for 15 months after his discharge. During this period, he was compliant with his antibiotic therapy, as well as had multiple routine blood cultures drawn, none of which showed any growth.

## 3. Discussion

*C. acnes* is a Gram-positive, nonmotile, non-spore-forming, commensal bacillus of human skin. It is most widely recognized as contributing to the pathogenesis of acne, while less appreciated in the pathogenesis of other conditions. The ability of *C. acnes* to form biofilms may be its most recognized virulence factor, as it can colonize artificial substrates [3]. It has been recently suggested that lysis of bacterial cells and release of cytoplasmic contents facilitate the formation of their biofilm [3]. Biofilm production may have been an important factor in the case presented, as the patient continued to have several positive blood cultures over the course of 4 years that were incompletely treated with antibiotics.

Achermann et al. suggests that a possible risk factor for hematogenous seeding of *C. acnes* and subsequent colonization of implanted devices are invasive procedures involving sebaceous gland-rich skin where *C. acnes* counts are the greatest [2]. Even with adequate topical sanitation techniques, studies have shown viable *C. acnes* skin recolonization of wound edges after 90–180 minutes, the point at which hematogenous seeding is possible. [2].

One study found blood cultures to be positive in only 62% of people with proven *C. acnes* infections [5]. In a case series by Sohail et al., 7 of 8 patients diagnosed with *C. acnes* endocarditis from 1967–2005 were men who ranged from 46 to 80 years of age. Two of these seven patients had positive lead cultures from either a PPM or implantable cardiac defibrillator. The remainder had some form of cardiac prosthesis [5, 6]. Our patient had characteristics consistent with those of the population in this study, and so his multiple episodes of symptomatic *C. acnes* bacteremia were only treated with short courses of a beta-lactam antibiotics before finally being diagnosed with an endocardial vegetation on old pacemaker leads.

Our study adds to the growing body of relatively new literature suggesting that *C. acnes* should be considered as more than just a skin contaminant when found in the blood of patients with implantable cardiac devices. Clinicians should have a low threshold to investigate these devices as a source of infection when *C. acnes* is isolated from the blood. Additionally, these patients should be treated aggressively with appropriate therapies including hardware removal, if possible. In the absence of hardware removal, long-term suppressive antibiotic therapy should be considered, as was decided by our patient, since inadequate treatment of *C. acnes* infective endocarditis can have devastating consequences. Although *C. acnes* endocarditis is rare, it has been demonstrated to be associated with abscess formation in up to 36% of cases. In cases of *C. acnes* bacteremia, mortality can occur in up to 5.9–16% of patients [7]. Clayton et al. identified several factors that contributed to more negative outcomes in patients with *C. acnes* endocarditis. These include an indolent clinical course, negative or delayed culture results, and the tendency to consider the organism as a skin contaminant, many of which occurred in our patient's disease course [8]. For our patient, the lower sensitivity of TTE for endocarditis was also an important factor contributing to delay in proper treatment.

Gomes et al. found that patients with retained pacemaker lead fragments following attempted transvenous lead extraction had a significantly increased risk of cardiac device infection compared to those who had complete removal of their leads (13.5% vs. 3%, *P*=0.001) [9]. Similar results were found by a recent propensity-matched analysis of the MEDIC trial [10]. According to the Heart Rhythm Society (HRS), the goal for extraction of a pacemaker in a patient with an implantable cardiac electronic device-related infection should be complete removal [11]. This recommendation stemmed from findings that one-third of patients with infection of retained leads ultimately required open heart surgery for infective endocarditis of the leads, despite antibiotics [12]. The American Association for Thoracic Surgery guidelines for treatment of patients with cardiac device-associated endocarditis with relapsing bacteremia despite appropriate antibiotic treatment also recommend surgical extraction (Grade IIa recommendation). [13].

Throughout the 15 months of therapy that we were able to follow, the patient had multiple routine blood cultures drawn that showed no growth, but further monitoring is necessary because of the long duration of cardiac device-related infections due to *C. acnes*. However, thus far, this is the first case to our knowledge in which indefinite suppressive antibiotics have been used successfully to control recurrent cardiac device-related infections when the pacemaker leads could not be completely removed.

## 4. Conclusions

*C. acnes* should be considered as a potential source of infection in patients with implanted medical devices and positive cultures. While often regarded as a blood culture contaminant, positive findings should not be immediately discounted due to the potential pathogenicity of the organism in this population. This bacterium is known to produce biofilms giving it a predilection for implanted medical devices and artificial surfaces. Echocardiography should be considered for diagnosis in patients with prosthetic cardiac valve and pacemakers. There should be a low threshold for transesophageal echocardiography in patients with multiple *C. acnes*-positive blood cultures, even if the transthoracic echocardiogram does not show signs of vegetations. Although unavailable in our case, histopathological and microbiologic studies of resected specimens are additional opportunities for reaching a potential diagnosis. Patients with persistently positive cultures will likely require surgical explantation of infected hardware to achieve cure. When surgical removal is not possible, suppressive antibiotic therapy should be considered.

*C. acnes* is an emerging, clinically relevant pathogen, especially when considering the rising rates of prosthetic surgical implants. In our patient, retained lead wires were the nidus for his endocarditis by this commensal organism. Persistently positive blood cultures in the setting of implantable cardiac devices should prompt a work-up for endocarditis, regardless of the bacterium. This case demonstrates how serious infections of implantable cardiac devices can be missed when pathogens of low-virulence, such as *C. acnes*, are overlooked as contaminants.

## Figures and Tables

**Table 1:** Overview of the circumstances surrounding each of the patients' *C. acnes*-positive blood cultures, including those from this presentation.

Positive blood culture set (time prior to final diagnosis of infective endocarditis)	Reason for presentation/pertinent findings at presentation	Location presented to	Results of blood culture drawn at presentation	Days after being drawn that blood cultures resulted positive/speciated *C.acnes*	Was the patient admitted after initial presentation?	Did the patient receive TTE evaluating for infective endocarditis due to this presentation?	Did the patient receive antibiotics therapy or other related infectious management due to this presentation?	Were there management changes after return of positive blood cultures?	Reasoning for management plan following return of positive blood cultures	Was there noted acknowledgement of previous positive blood cultures?	Did the patient ever receive TEE in the evaluation of these blood culture results?
#1 (4 years prior)	Fevers, rigors, and leukocytosis	Emergency department	*C.acnes* in 2/2 anaerobic bottles	3/6	No	No	No	No changes in management	When the patient was followed up with 7 days after presentation, he had since been assessed by an outside physician and was currently asymptomatic. Positive blood cultures were not further addressed	n/a	No

#2 (3 years, 11 months prior)	Fevers and leukocytosis	Emergency department	*C.acnes* in 2/2 anaerobic bottles	4/9	No	No	Discharged with course of levofloxacin and out-patient follow-up with infectious disease	No changes in management	The patient was already taking antibiotics and had follow-up with infectious disease scheduled to determine etiology. Outcome of follow-up unknown, though the patient did not have another positive blood culture for 3 years after this admission	Yes, which helped guide recommendation to see out-patient infectious disease.	No

#3 (10 months prior)	Fevers, dental pain, and leukocytosis	Emergency department	*C.acnes* in 2/2 anaerobic bottles	5/7	No	No	Discharged with 10 days of course of penicillin and out-patient follow-up with oral surgery	No changes in management	Patient was already taking antibiotics and was planning to follow-up with oral surgery for a presumed dental infection	No	No

#4 (6 months prior)	Pleuritic chest pain, fevers, leukocytosis, and a chest X-ray showing left-sided infiltrate and a large left-sided pleural effusion	Emergency department	*C.acnes* in 1/2 anaerobic bottles	5/8	Yes	Yes, which was negative for infective endocarditis	Started empiric board-spectrum antibiotics at presentation for presumed pneumonia	Infectious disease consulted, a TTE was ordered, and repeat blood cultures were drawn	To evaluate for etiology of recurrent *C.acnes* bacteremia. No further evaluation was pursued after negative TTE, 2 subsequent sets of negative blood cultures, and the patient's symptomatic improvement. It was stated that the positive blood culture was likely the result of contamination, as only1/2 anaerobic bottles grew out *C.acnes* and the subsequent blood cultures were all negative	Yes, acknowledged “previous C.acnes-positive blood cultures of unclear etiology”.	No

#5 (1 month prior)	Undocumented	Skilled nursing facility	*C.acnes* in 2/2 anaerobic bottles	5/7	No	No	No	No changes in management	No noted actions taken after return of positive blood cultures, though the patient presented to the ED the following day	No	No

#6 (3 weeks prior)	Fevers, shortness of breath, and an erythematous, tender right chest fluctuance	Emergency department	*C.acnes* in 1/2 anaerobic bottles	4/6	No	No	No	No changes in management	Believed positive blood culture to be due to a contaminant	Yes, acknowledged “positive blood cultures from prior”.	No

#7 (1 week prior)	hypoglycemia, fevers, and an erythematous, tender right chest fluctuance	Emergency department	*C.acnes* in 2/2 anaerobic bottles	4/6	Yes	Yes, which was negative for infective endocarditis	Started empiric board-spectrum antibiotics at presentation for presumed sepsis	TEE was ordered	Suspicion for infection endocarditis despite negative TTE	Yes, which help guide decision to obtain TEE.	Yes, which showed vegetations on multiple pacemaker leads, the largest being 1 cm.
